# Supplementary material for: Theory on the rate equations of Michaelis-Menten type enzyme kinetics with competitive inhibition
Source: PLoS One. 2024 Jul 18;19(7):e0302679. doi: 10.1371/journal.pone.0302679 (PMC11257316; doi:10.1371/journal.pone.0302679)
Supplement: S1 Appendix — (DOCX) [file pone.0302679.s001.docx]

**Appendix A**

Consider the following set of coupled linear second order ODEs.

$\frac{d^{2}M}{d\tau^{2}}+\frac{\alpha_{S}}{\eta_{S}}\frac{dM}{d\tau}+\frac{\varepsilon_{S}}{\eta_{S}}M-\frac{1}{{\eta_{S}}^{3}}+\frac{{\eta_{I}}^{2}}{\rho{\eta_{S}}^{3}}\frac{dN}{d\tau}\cong0;\left[ M \right]_{\tau=0}=0;\left[ \frac{dM}{d\tau} \right]_{\tau=0}=0$ . [A1]

$\frac{d^{2}N}{d\tau^{2}}+\frac{{\rho\alpha}_{I}}{\eta_{I}}\frac{dN}{d\tau}+\frac{{\varepsilon_{I}\rho}^{2}N}{\eta_{I}}-\frac{\rho^{2}}{{\eta_{I}}^{3}}+\frac{\rho^{2}{\eta_{s}}^{2}}{{\eta_{I}}^{3}}\frac{dM}{d\tau}\cong0;\left[ N \right]_{\tau=0}=0;\left[ \frac{dN}{d\tau} \right]_{\tau=0}=0$. [A2]

This system can be uncoupled as follows. Upon differentiating **Eqs. A2** with respect to τ, one arrives at the following third order ODE.

$\frac{d^{3}N}{d\tau^{3}}+\frac{{\rho\alpha}_{I}}{\eta_{I}}\frac{d^{2}N}{d\tau^{2}}+\frac{{\varepsilon_{I}\rho}^{2}}{\eta_{I}}\frac{dN}{d\tau}+\frac{\rho^{2}{\eta_{s}}^{2}}{{\eta_{I}}^{3}}\frac{d^{2}M}{d\tau^{2}}\cong0$. [A3]

From **Eqs. A1**, one can derive the following expression.

$\frac{dN}{d\tau}=-\frac{\rho{\eta_{S}}^{3}}{{\eta_{I}}^{2}}\left( \frac{d^{2}M}{d\tau^{2}}+\frac{\alpha_{S}}{\eta_{S}}\frac{dM}{d\tau}+\frac{\varepsilon_{S}}{\eta_{S}}M-\frac{1}{{\eta_{S}}^{3}} \right)$. [A4]

By repeated differentiation of **Eq. A4** with respect to τ, we obtain the following relationships.

$\frac{d^{2}N}{d\tau^{2}}=-\frac{\rho{\eta_{S}}^{3}}{{\eta_{I}}^{2}}\left( \frac{d^{3}M}{d\tau^{3}}+\frac{\alpha_{S}}{\eta_{S}}\frac{d^{2}M}{d\tau^{2}}+\frac{\varepsilon_{S}}{\eta_{S}}\frac{dM}{d\tau} \right)$ . [A5]

$\frac{d^{3}N}{d\tau^{3}}=-\frac{\rho{\eta_{S}}^{3}}{{\eta_{I}}^{2}}\left( \frac{d^{4}M}{d\tau^{4}}+\frac{\alpha_{S}}{\eta_{S}}\frac{d^{3}M}{d\tau^{3}}+\frac{\varepsilon_{S}}{\eta_{S}}\frac{d^{2}M}{d\tau^{2}} \right)$. [A6]

Upon substituting **Eqs. A4-A6** into **Eq.** **A3**, one obtains the following uncoupled fourth order ODE corresponding to (M, τ) space.

$\frac{d^{4}M}{d\tau^{4}}+a\frac{d^{3}M}{d\tau^{3}}+b\frac{d^{2}M}{d\tau^{2}}+c\frac{dM}{d\tau}+hM-\frac{{\varepsilon_{I}\rho}^{2}}{{\eta_{S}}^{3}\eta_{I}}\cong0$ . [A7]

In this equation, various terms *a*, *b*, *c* and h are defined as follows.

$a=\left( \frac{\alpha_{S}}{\eta_{S}}+\frac{{\rho\alpha}_{I}}{\eta_{I}} \right);b=\left( \frac{\varepsilon_{S}}{\eta_{S}}+\frac{\rho\left( {\alpha_{S}\alpha}_{I}-1 \right)}{\eta_{I}\eta_{S}}+\frac{{\varepsilon_{I}\rho}^{2}}{\eta_{I}} \right);c=\frac{\rho}{\eta_{I}}\left( \frac{\alpha_{I}\varepsilon_{S}+{\varepsilon_{I}\rho\alpha}_{S}}{\eta_{S}} \right);h=\frac{{\varepsilon_{I}\varepsilon_{S}\rho}^{2}}{{\eta_{S}\eta}_{I}}$. [A8]

The first two initial conditions associated with **Eq. A7** are as follows.

$\left[ M \right]_{\tau=0}=0;\left[ \frac{dM}{d\tau} \right]_{\tau=0}=0$. [A9]

Other two initial conditions directly follow from the initial conditions corresponding to N.

$\left[ \frac{dN}{d\tau} \right]_{\tau=0}=\left[ \frac{d^{2}M}{d\tau^{2}}+\frac{\alpha_{S}}{\eta_{S}}\frac{dM}{d\tau}+\frac{\varepsilon_{S}}{\eta_{S}}M-\frac{1}{{\eta_{S}}^{3}} \right]_{\tau=0}=0$. [A10]

$\left[ N \right]_{\tau=0}=\left[ \frac{d^{3}M}{d\tau^{3}}+\frac{\alpha_{S}}{\eta_{S}}\frac{d^{2}M}{d\tau^{2}}+\left( \frac{\varepsilon_{S}}{\eta_{S}}+\frac{\rho^{2}{\eta_{s}}^{2}}{{\eta_{I}}^{3}} \right)\frac{dM}{d\tau}-\frac{\rho^{2}}{{\eta_{I}}^{3}} \right]_{\tau=0}=0$. [A11]

Upon obtaining the solution for the (M, τ) space, one can directly obtain the expression corresponding to the (N, τ) space as follows.

$N=\frac{\rho{\eta_{S}}^{3}}{{\eta_{I}}^{2}}\int_{0}^{\tau} \left( \frac{d^{2}M}{d\tau^{2}}+\frac{\alpha_{S}}{\eta_{S}}\frac{dM}{d\tau}+\frac{\varepsilon_{S}}{\eta_{S}}M-\frac{1}{{\eta_{S}}^{3}} \right)d\tau.$ [A12]

Similar to **Eqs. A3-A12**, one can also derive the following solution set. From **Eqs. A2**, one can derive the following expression.

$\frac{dM}{d\tau}=-\frac{{\eta_{I}}^{3}}{\rho^{2}{\eta_{s}}^{2}}\left( \frac{d^{2}N}{d\tau^{2}}+\frac{{\rho\alpha}_{I}}{\eta_{I}}\frac{dN}{d\tau}+\frac{{\varepsilon_{I}\rho}^{2}N}{\eta_{I}}-\frac{\rho^{2}}{{\eta_{I}}^{3}} \right)$. [A13]

By repeated differentiation of **Eq. A13** with respect to τ, we obtain the following relationships.

$\frac{d^{2}M}{d\tau^{2}}=-\frac{{\eta_{I}}^{3}}{\rho^{2}{\eta_{s}}^{2}}\left( \frac{d^{3}N}{d\tau^{3}}+\frac{{\rho\alpha}_{I}}{\eta_{I}}\frac{d^{2}N}{d\tau^{2}}+\frac{{\varepsilon_{I}\rho}^{2}}{\eta_{I}}\frac{dN}{d\tau} \right)$. [A14]

$\frac{d^{3}M}{d\tau^{3}}=-\frac{{\eta_{I}}^{3}}{\rho^{2}{\eta_{s}}^{2}}\left( \frac{d^{4}N}{d\tau^{4}}+\frac{{\rho\alpha}_{I}}{\eta_{I}}\frac{d^{3}N}{d\tau^{3}}+\frac{{\varepsilon_{I}\rho}^{2}}{\eta_{I}}\frac{d^{2}N}{d\tau^{2}} \right)$. [A15]

Upon differentiating **Eq. A1** with respect to τ, one obtains the following expression.

$\frac{d^{3}M}{d\tau^{3}}+\frac{\alpha_{S}}{\eta_{S}}\frac{d^{2}M}{d\tau^{2}}+\frac{\varepsilon_{S}}{\eta_{S}}\frac{dM}{d\tau}+\frac{{\eta_{I}}^{2}}{\rho{\eta_{S}}^{3}}\frac{d^{2}N}{d\tau^{2}}$. [A16]

Upon substituting the derivatives from **Eqs. A13-A15** into **Eq.** **A16**, one obtains the following uncoupled fourth order ODE corresponding to (N, τ) space.

$\frac{d^{4}N}{d\tau^{4}}+r\frac{d^{3}N}{d\tau^{3}}+g\frac{d^{2}N}{d\tau^{2}}+m\frac{dN}{d\tau}+zN-\frac{\varepsilon_{S}}{\eta_{S}}\frac{\rho^{2}}{{\eta_{I}}^{3}}\cong0$ . [A17]

Here the parameters r, *g*, m, and z are defined as follows.

$r=\left( \frac{{\rho\alpha}_{I}}{\eta_{I}}+\frac{\alpha_{S}}{\eta_{S}} \right);g=\left( \frac{{\varepsilon_{I}\rho}^{2}}{\eta_{I}}+\frac{\rho\left( {\alpha_{S}\alpha}_{I}-1 \right)}{\eta_{S}\eta_{I}}+\frac{\varepsilon_{S}}{\eta_{S}} \right);m=\left( \frac{\alpha_{S}{\varepsilon_{I}\rho}^{2}-{\rho\varepsilon_{S}\alpha}_{I}}{{\eta_{S}\eta}_{I}} \right);z=\frac{{\varepsilon_{I}\rho}^{2}\varepsilon_{S}}{{\eta_{I}\eta}_{S}}$. [A18]

Upon obtaining the solution for the (N, τ) space one can directly obtain the expression corresponding to the (M, τ) space as follows.

$M=\frac{{\eta_{I}}^{3}}{\rho^{2}{\eta_{s}}^{2}}\int_{0}^{\tau} \left( \frac{d^{2}N}{d\tau^{2}}+\frac{{\rho\alpha}_{I}}{\eta_{I}}\frac{dN}{d\tau}+\frac{{\varepsilon_{I}\rho}^{2}N}{\eta_{I}}-\frac{\rho^{2}}{{\eta_{I}}^{3}} \right)d\tau$. [A19]

The initial conditions corresponding to the fourth order uncoupled ODE given by **Eqs. A17** can be written as follows.

$\left[ N \right]_{\tau=0}=0;\left[ \frac{dN}{d\tau} \right]_{\tau=0}=0$. [A20]

Other two initial conditions directly follow from the initial conditions corresponding to M.

$\left[ \frac{dM}{d\tau} \right]_{\tau=0}=\left[ \frac{d^{2}N}{d\tau^{2}}+\frac{{\rho\alpha}_{I}}{\eta_{I}}\frac{dN}{d\tau}+\frac{{\varepsilon_{I}\rho}^{2}N}{\eta_{I}}-\frac{\rho^{2}}{{\eta_{I}}^{3}} \right]_{\tau=0}=0$. [A21]

$\left[ M \right]_{\tau=0}=\left[ \frac{d^{3}N}{d\tau^{3}}+\frac{{\rho\alpha}_{I}}{\eta_{I}}\frac{d^{2}N}{d\tau^{2}}+\left( \frac{{\varepsilon_{I}\rho}^{2}}{\eta_{I}}+\frac{{\eta_{I}}^{2}}{\rho{\eta_{S}}^{3}} \right)\frac{dN}{d\tau}-\frac{1}{{\eta_{S}}^{3}} \right]_{\tau=0}=0$ . [A22]

Solution to **Eqs. A1**-**A2** can be written as follows.

$M=\left( \frac{1}{{{\varepsilon_{S}\eta}_{S}}^{2}}+C_{1}exp\left( r_{1}\tau\right)+C_{2}exp\left( r_{2}\tau\right)+C_{3}exp\left( r_{3}\tau\right)+C_{4}exp\left( r_{4}\tau\right) \right)$. [A23]

From this equation, N can be obtained using **Eq. A12**. Here r_1_, r_2_, r_3_, and r_4_ are the roots of the following fourth degree polynomial in r.

$\eta_{I}\eta_{S}r^{4}+\left( {\rho\alpha}_{I}\eta_{S} +\alpha_{S}\eta_{I} \right)r^{3}+\left( \eta_{S}\varepsilon_{I}\rho^{2}+ {\rho\alpha}_{I}\alpha_{S} +\eta_{I}\varepsilon_{S}-\eta_{S}\rho\right)r^{2}+\left( \alpha_{S}\varepsilon_{I}\rho^{2}+{\rho\alpha}_{I}\varepsilon_{S} \right)r+\rho^{2}\varepsilon_{I}\varepsilon_{S}=0$. [A24]

Upon using the initial conditions given by **Eqs. A9**-**11**, one can obtain the expressions for various constant terms C_1_-C_4_ in **Eqs. A23** as follows.

$C_{1}=\frac{\left( r_{2}r_{3}r_{4}{\eta_{S}}^{2}- \varepsilon_{S}\left( r_{2} + r_{3} + r_{4} \right)\eta_{S} - \varepsilon_{S}\alpha_{S} \right){\eta_{I}}^{3}+ {\eta_{S}}^{4}\varepsilon_{S}\rho^{2}}{\left( r_{1} - r_{4} \right)\left( r_{1} - r_{3} \right){\eta_{I}}^{3}\left( r_{1} -r_{2} \right){\eta_{S}}^{4}\varepsilon_{S}}$. [A25]

$C_{2}=\frac{\left( -r_{1}r_{3}r_{4}{\eta_{S}}^{2}+ \varepsilon_{S}\left( r_{1} + r_{3} + r_{4} \right)\eta_{S} + \varepsilon_{S}\alpha_{S} \right){\eta_{I}}^{3}- {\eta_{S}}^{4}\varepsilon_{S}\rho^{2}}{{\eta_{S}}^{4}\varepsilon_{S}\left( r_{1} -r_{2} \right)\left( r2 - r_{4} \right){\eta_{I}}^{3}\left( r_{2} - r_{3} \right)}$. [A26]

$C_{3}=\frac{\left( r_{1}r_{2}r_{4}{\eta_{S}}^{2}- \varepsilon_{S}\left( r_{1} + r_{2} + r_{4} \right)\eta_{S}- \varepsilon_{S}\alpha_{S} \right){\eta_{I}}^{3}+ {\eta_{S}}^{4}\varepsilon_{S}\rho^{2}}{{\eta_{S}}^{4}\varepsilon_{S}\left( r_{2} - r_{3} \right)\left( r_{1} - r_{3} \right)\left( r_{3} -r_{4} \right){\eta_{I}}^{3}}$. [A27]

$C_{4}=\frac{\left( -r_{1}r_{2}r_{3}{\eta_{S}}^{2}+ \varepsilon_{S}*\left( r_{1} + r_{2} + r_{3} \right)\eta_{S} + \varepsilon_{S}\alpha_{S} \right){\eta_{I}}^{3}- {\eta_{S}}^{4}\varepsilon_{S}\rho^{2}}{{\eta_{I}}^{3}{\eta_{S}}^{4}\varepsilon_{S}\left( r_{3} - r_{4} \right)\left( r_{2} - r_{4} \right)\left( r_{1} - r_{4} \right)}$. [A28]

Using the transformations $\left( P,V \right)={{\varepsilon_{S}\eta}_{S}}^{2}\left( M,F \right)$ and ${{{\left( Q,U \right)=\varepsilon}_{I}\eta}_{I}}^{2}\left( N,G \right)$ one can revert back to the original dynamical variables V, U, P and Q. Subsequently one finds that S = 1 – V – P and I = 1 – U/ρ – Q. Here the dynamical variables (S, I, V, U, P, Q) are all expressed over (V, S, I), (U, S, I), (V, P, Q) and (U, P, Q) spaces in the parametric form where τ acts as the parameter.

**Appendix B**

Let us consider the following nonlinear first order ODE along with the initial condition.

$F\frac{dF}{dM}+aF-b\cong0;\left[ F \right]_{M=0}=0$. [B1]

Here $a=\left( \frac{\alpha_{S}\alpha_{I}-1}{\eta_{S}\alpha_{I}} \right)$ and $b=\left( \frac{\alpha_{I}-1}{{{\eta_{S}}^{3}\alpha}_{I}} \right)$. Upon solving **Eq. B1** implicitly and reverting back to (V, P) space using the transformation $\left( V,P \right)=\varepsilon_{S}\eta_{S}^{2}\left( F,M \right)$, one obtains the following solution.

$\frac{P}{\varepsilon_{S}\eta_{S}^{2}}+\frac{V}{a\varepsilon_{S}\eta_{S}^{2}}+\frac{b}{a^{2}}ln\left( 1-\frac{a}{b\varepsilon_{S}\eta_{S}^{2}}V \right)=0$. [B2]

Upon substitution of the conservation law, P = 1 – V – S in **Eq. B2** and after few manipulations and rearrangements after adding $\left( a-1 \right)$ term both sides of equation, one finds the following implicit solution corresponding to the (V, S) space.

$-\frac{a\left( a-1 \right)}{bc}V+\left( a-1 \right)+ln\left( -\frac{a\left( a-1 \right)}{bc}V+\left( a-1 \right) \right)=- \left( \frac{1-S}{c} \right)\frac{a^{2}}{bc}+ln\left( a-1 \right)+\left( a-1 \right)$ . [B3]

Here $c=\varepsilon_{S}\eta_{S}^{2}$. Upon exponentiating both sides of **Eq. B3**, one arrives at the form $Wexp\left( W \right)=Z$ as follows.

$\left( \left( a-1 \right)-\frac{a\left( a-1 \right)}{bc}V \right)exp\left( \left( a-1 \right)-\frac{a\left( a-1 \right)}{bc}V \right)=\left( a-1 \right)exp\left( - \left( \frac{1-S}{c} \right)\frac{a^{2}}{bc}+\left( a-1 \right) \right).$ [B4]

Solution to **Eq. B4** can be written in terms of Lambert W function as follows.

$V\cong\frac{bc}{a}\left( 1-\frac{1}{\left( a-1 \right)}W\left( \left( a-1 \right)exp\left( -\left( \frac{1-S}{c} \right)\frac{a^{2}}{bc}+\left( a-1 \right) \right) \right) \right)$. [B5]

Upon inserting the corresponding values of a, b and c in **Eq. B5**, one finally arrives at the following approximation in the pre-steady state regime of the (V, S) space.

$V\cong\frac{\varepsilon_{S}\left( \alpha_{I}-1 \right)}{\left( {\alpha_{S}\alpha}_{I}-1 \right)}\left( 1-\frac{1}{\left( a - 1 \right)}W\left( \left( a - 1 \right)exp\left( \left( a - 1 \right)+\frac{a^{2}}{b\varepsilon_{S}{\eta_{S}}^{2}}\left( S-1 \right) \right) \right) \right)$ . [B6]

Let us consider the following properties of the Lambert’s W(Z) function.

$exp\left( W \right)=Z/W; \frac{dW}{dZ}exp\left( W \right)\left( 1+W \right)=1; \frac{dW}{dZ}=\frac{1}{exp\left( W \right)\left( 1+W \right)}=\frac{W}{Z\left( 1+W \right)}$. [B7]

Upon expanding the right-hand side of **Eq. B5** using **Eq. B7** in a Taylor series around S = 1, noting that $W\left( Zexp\left( Z \right) \right)=Z$, one finds the following series.

$V\cong1-S-{\left( S-1 \right)^{2}}/{2bc}+O\left( \left( S-1 \right)^{3} \right)$. [B8]

Since the amount of product building up in the pre-steady state regime will be negligible in most of the scenarios, one can ignore the second order terms in **Eq. B8** and use the pre-steady state approximation as $V\cong1-S$ in the (V, S) space.
